# Characterization of a Pure Isolate from *Atalantia ceylanica* Leaves and Its Biological Activities

**DOI:** 10.3390/biom16050663

**Published:** 2026-04-30

**Authors:** Savani Ulpathakumbura, Rasika Gunarathne, Lalith Jayasinghe, Yoshinori Fujimoto, Nazrim Marikkar, Johnson Liu, Ji He, Jun Lu

**Affiliations:** 1 National Institute of Fundamental Studies, Hanthana Road, Kandy 20000, Sri Lanka; buthsara.ul@nifs.ac.lk (S.U.);; 2 Auckland Bioengineering Institute, University of Auckland, Auckland 1142, New Zealand; 3 School of Agriculture, Meiji University, Kawasaki 214-8571, Japan; 4 School of Biomedical Sciences, University of New South Wales, Sydney, NSW 2052, Australia; 5 Future Food and Agriculture Department, Yangtze Delta Region Institute of Tsinghua University, Jiaxing 314006, China; heji@tsinghua-zj.edu.cn; 6 School of Chemical Sciences, University of Auckland, Auckland 1142, New Zealand

**Keywords:** *Atalantia ceylanica*, Yaki naran leaves, diabetic remedies, NMR structure elucidation

## Abstract

*Atalantia ceylanica*, locally known as Yaki naran (YK), is a native plant of Sri Lanka, growing commonly in the dry and wet–intermediate zones. In this study, powdered samples of Yaki naran (YK) were sequentially extracted using hexane, ethyl acetate (EtOAc), and methanol (MeOH). The resulting extracts were assessed for total phenolic content, antioxidant potentials, and in vitro α-amylase, α-glucosidase, and lipase inhibitory activities using relevant assays. The crude extracts were then subjected to separation and purification by column chromatography and preparative thin-layer chromatography. Although twelve compounds were obtained from the three crude extracts, only three had sufficient yields to proceed. Out of the three pure isolates, compound SAC 4 was identified as 2,4-di-tert-butylphenol, a phenolic compound, by using ^1^H and ^13^C NMR data and FTIR spectroscopic data, followed by evaluation of bioactivities such as antioxidant properties, enzyme inhibitory assays, etc. Based on the results of the bioassays, compound SAC 4 was identified to show strong α-glucosidase inhibitory activity, moderate antioxidant activity, and lipase inhibitory activity.

## 1. Introduction

Sri Lanka is one of the most diverse biological hotspots in Asia, with nearly 30% of its land area covered by forests [1]. The ecological prosperity of the country is further reflected in hosting approximately 4143 plant species classified under 1522 genera and 214 families [2]. According to a recent survey by Rajakaruna et al. [3], Sri Lanka has a total number of 1476 plant species, belonging to 194 families. Several of these plants have played significant roles, serving as a source of food and remedies against several ailments. Plant materials, such as seeds of *Syzygium cumini*, leaves of *Adhathoda vasica*, roots of *Oryza sativa*, and fruits of *Momordica dioica* are notable examples used as traditional remedies for control of diabetes [4]. A recent study by Uyangoda & Munasinghe [5] highlighted the therapeutic potential of *Ageratum conyzoides*, *Artocarpus gomezianus*, *Euphorbia hirta*, *Plectranthus zeylanicus*, and *Piper sarmentosu* against diabetes, inflammation, cancers, etc. In another study, fifteen traditional Sri Lankan plants were found to exhibit therapeutic potential against various skin disorders [6]. The strong reliance on plant-derived medicines underscores the clinical significance of Sri Lankan botanicals and highlights the need for continued scientific exploration of medicinal plants. Accordingly, recent efforts have focused on five edible leafy plants—*Premna procumbens* Moon, *Polyscias scutellaria* Fosberg, *Stevia rebaudiana* Bertoni, *Memecylon umbellatum*, and *Atalantia ceylanica*—to investigate their anti-diabetic and anti-obesity potential through α-glucosidase, α-amylase, and lipase inhibitory activities [7,8]. Among these, leaves of *A. ceylanica* demonstrated notable antioxidant capacity and strong α-glucosidase inhibitory activity, prompting further interest in identifying its bioactive constituents for diabetes and obesity management.

*A. ceylanica*, belonging to the family Rutaceae, is native to Sri Lanka and is locally known as *Yaki naran* in Sinhala. It is commonly found in the dry and intermediate zones growing as a densely branched shrub. The leaves are ovate, ovate–lanceolate, or elliptical in shape and are used in culinary applications, including the preparation of herbal porridge [9,10] where the bark, roots, and flowers are reported to possess various medicinal properties. Fernando & Soysa [11] noted that leaf extracts are used in the preparation of pills for treating catarrh, bronchitis, and other chest-related complications. The study also reported the use of leaf extracts of *A. ceylanica* in the treatment of liver diseases, while the roots have been used for treating ague. However, the study did not provide detailed information on the phytochemical constituents responsible for these bioactivities. In a recent screening of five leafy plants, *A. ceylanica* emerged as a species exhibiting multiple biological activities [7]. Given its significant medicinal potential, the present study aimed to fractionate and purify leaf extracts of this plant to isolate the secondary metabolites responsible for these activities. Accordingly, the hexane, ethyl acetate (EtOAc), and methanol fractions were subjected to column chromatography to isolate pure compounds, which were subsequently characterized through structural elucidation and evaluated for their biological activities.

## 2. Materials and Methods

### 2.1. Plant Materials

Mature leaves of Yaki naran (YK) were collected from the North Central Province (Dambulla area) of Sri Lanka between March and May 2021. The plant material was authenticated by a botanist, and voucher specimens were deposited at Popham’s Arboretum of NIFS in Dambulla, Sri Lanka. The leaves were washed with running tap water and then dried in a forced-air drying oven (model: BOV-V230F, Biobase, Jinan, Shandong, China) at 55 °C for 8–10 h. The dried leaves were then ground into a fine powder and stored at 4 °C until further analysis.

### 2.2. Reagents and Instruments

The enzymes α-amylase (porcine pancreatic), α-glucosidase, and porcine pancreatic lipase were purchased from Sigma-Aldrich, Inc., St. Louis, MO, USA. All other chemicals and reagents used in the assays were of analytical grade, unless otherwise specified. UV absorbance measurements were performed using a microplate reader (Synergy HTX Biotek Multimode reader, Biotek Instruments, Inc., Winooski, VT, USA).

### 2.3. Preparation of Crude Extracts

Powdered YK leaves (1 kg) were sequentially extracted with hexane, EtOAc, and MeOH using ultrasonication at 53 kHz frequency (Rocker ultrasonic cleaner, model: Soner 206H, Rose Scientific Ltd., Edmonton, AB, Canada) for 30 min at 30 °C. The extraction process was repeated three times for each solvent. The resulting extracts were concentrated under reduced pressure using a rotary evaporator (Laborota 4000, Heidolph Instrument GmbH & Co KG, Schwabach, Germany) and subsequently vacuum-dried (Heraeus VT6025 Vacuumthermvacuum drying oven, Heraeus Holding GmbH, Hanau, Germany) at room temperature for 3–4 h. The dried crude extracts were stored at −18 °C until further analysis.

### 2.4. Sub-Fractionation of Crude Extracts

Fractionation of the crude extracts was conducted according to the method described by Dissanayake [9]. Briefly, 4 g of the hexane extract was subjected to gravity column chromatography using a glass column (3 cm diameter) packed with silica gel (70–230 mesh) to a height of approximately 4 inches, with dichloromethane (DCM) as the initial mobile phase. The crude extract was loaded onto the column as a dry slurry prepared with silica gel. Elution was performed using DCM and MeOH in increasing order of polarity. A total of eight fractions were collected and monitored using thin-layer chromatography (TLC). A similar procedure was followed for the EtOAc (4.7 g) and MeOH (6.5 g) extracts, yielding seven and nine fractions, respectively. Based on similarities in TLC profiles observed under UV light (254 nm), the 24 fractions were pooled into seven combined fractions. These fractions were further purified to isolate twelve pure compounds, designated SAC 1–SAC 12. The yields of SAC 4, SAC 7, and SAC 9 were 7.4 mg, 4.4 mg, and 3.1 mg, respectively.

### 2.5. Structure Elucidation of Compound SAC 4

The structure of SAC 4 was elucidated using 1D (^1^H and ^13^C) and 2D (including COSY, HMBC, and NOE) NMR spectroscopy. NMR experiments were carried out using a Bruker DRX 500 spectrometer (Bruker Biospin GmbH, Bremen, Germany) (500 MHz for ^1^H and 125 MHz for ^13^C), dissolving samples in CDCl_3_ as the solvent. To confirm the functional groups present in the compound, FTIR spectroscopy was performed following the method described by Mittal et al. [12]. Briefly, approximately 1.5 mg of the pure isolate was mixed with 90 mg of KBr (FT-IR grade, ≥99% trace metals basis, Sigma Aldrich Inc., St. Louis, MO, USA) and compressed into a pellet using a hydraulic press. The spectra were recorded using a Nicolet iS50 FTIR spectrometer (Thermo Nicolet, Madison, WI, USA) equipped with a deuterated triglycine sulfate (DTGS) detector and a KBr beam splitter.

### 2.6. Bioactivity of Compound SAC 4

#### 2.6.1. DPPH Radical Scavenging Activity

The antioxidant activity, in terms of DPPH radical scavenging activity, was determined according to the method described by Ulpathakumbura et al. [7]. A concentration series (0.78 to 100 mg L^−1^) was prepared in MeOH. A 150 µL aliquot of the sample was mixed with 60 µL of 0.3 mM DPPH solution in a 96-well microplate and then incubated in the dark at room temperature for 30 min. The absorbance was recorded at 517 nm using a plate reader (Synergy HTX Biotek Multimode reader, Biotek instruments, Inc., Winooski, VT, USA). The assay included ascorbic acid as the positive control reference. The percentage of radical scavenging activity (RSA%) was calculated using the following equation, while the IC_50_ values were determined graphically by plotting RSA% against the respective sample concentrations:

$$RSA\%=\frac{\delta {A\;}_{\mathrm{control}}-\delta A_{\mathrm{sample}}}{\delta {A\;}_{\mathrm{control}}}\times 100$$

where $\delta {A\;}_{\mathrm{control}}\;={\mathrm{Absorbance}\;}_{\mathrm{control}}-{\mathrm{Absorbance}\;}_{\mathrm{control}\;\mathrm{blank}},$ $\delta A_{\mathrm{sample}}={\mathrm{Absorbance}\;}_{\mathrm{sample}}-{\mathrm{Absorbance}\;}_{\mathrm{sample}\;\mathrm{blank}}$.

#### 2.6.2. Ferric Reducing Antioxidant Power (FRAP)

A FRAP assay was carried out following the method described by Abubakar et al. [13]. Initially, a 100 mg L^−1^ sample solution was prepared by dissolving the pure compound in distilled water. A 50 µL aliquot of sample solution was then mixed with 200 µL of FRAP reagent and allowed to stand at room temperature for 4 min. The absorbance was measured at 593 nm using a plate reader (Synergy HTX Biotek Multimode reader, Biotek instruments, Inc., Winooski, VT, USA), and the results were expressed as µmole of FeSO_4_ per g of crude extract. Ascorbic acid was included as the positive control in this assay.

#### 2.6.3. ABTS^+^ Radical Scavenging Activity

The ABTS assay was performed according to the method described by Ulpathakumbura et al. [7]. A 100 mg L^−1^ sample solution was initially prepared by dissolving the pure compound in distilled water. A 50 µL aliquot of the sample solution was mixed with 150 µL of ABTS working solution and incubated at room temperature for 10 min. The absorbance was then measured at 734 nm using a microplate reader. Ascorbic acid was included as the positive control in the experiment. The results were expressed as µmole Trolox equivalents per gram of crude extract.

#### 2.6.4. α-Amylase Inhibitory Activity

α-Amylase inhibitory activity of pure isolate SAC 4 was performed according to the methods described by Ulpathakumbura et al. [7] and Nickavar et al. [14]. Briefly, samples were dissolved in distilled water to prepare a concentration series ranging from 100 to 1000 mg L^−1^. Then, an aliquot of 50 µL from each sample solution was combined with an equal amount of α-amylase enzyme solution (2 mg mL^−1^). The mixture was incubated for 30 min at room temperature. Following this, a 100 µL portion of 1% starch solution was added, and the mixture was allowed to process further for another 10 min at room temperature. Subsequently, 100 µL of DNSA reagent was added to the reaction mixture, which was then incubated in a water bath at 85 °C for 15 min. The mixture was then cooled down to room temperature and diluted with 900 µL of distilled water.

Afterwards, a 200 µL aliquot was transferred into the 96-well microplate, and the absorbance was recorded at 540 nm. Acarbose (Glucobay tablet) was used as the positive control in this assay. The percentage of enzyme inhibition was calculated using the following equation, and the IC_50_ values were calculated by plotting the inhibition percentage against the respective sample concentrations:

$$Percentage\;\alpha -amylase\;inhibition=\frac{\delta {A\;}_{\mathrm{control}}-\delta A_{\mathrm{sample}}}{\delta {A\;}_{\mathrm{control}}}\times 100$$

where $\delta {A\;}_{\mathrm{control}}={\mathrm{Absorbance}\;}_{\mathrm{control}}-{\mathrm{Absorbance}\;}_{\mathrm{control}\;\mathrm{blank}},$ $\delta A_{\mathrm{sample}}={\mathrm{Absorbance}\;}_{\mathrm{sample}}-{\mathrm{Absorbance}\;}_{\mathrm{sample}\;\mathrm{blank}}$.

#### 2.6.5. α-Glucosidase Inhibitory Activity

The α-glucosidase inhibitory activity of the pure isolate (SAC4) was determined according to the method described by Alakolanga et al. [15]. A concentration series (3.91–1000 mg L^−1^) was prepared by dissolving the pure compound in distilled water. Briefly, 100 µL of 30 mM phosphate buffer (pH 6.5) was added to each well of the 96-well microplate, followed by 25 µL of the sample solution. Subsequently, 25 µL of α-glucosidase enzyme solution was added, and the mixture was incubated at 37 °C for 5 min. Thereafter, 50 µL of pNPG solution (0.8 mg mL^−1^) was added, and the reaction mixture was further incubated at 37 °C for 30 min. Acarbose (Glucobay tablet) was used as the positive control. Absorbance was measured at 410 nm using a microplate reader. The percentage of α-glucosidase inhibition was calculated using a standard equation, and IC_50_ values were determined graphically by plotting the inhibition percentage against the sample concentration:

$$Percentage\;\alpha -glucosidase\;inhibition=\frac{\delta {A\;}_{\mathrm{control}}-\delta A_{\mathrm{sample}}}{\delta {A\;}_{\mathrm{control}}}\times 100$$

where $\delta {A\;}_{\mathrm{control}}={\mathrm{Absorbance}\;}_{\mathrm{control}}-{\mathrm{Absorbance}\;}_{\mathrm{control}\;\mathrm{blank}},$ $\delta A_{\mathrm{sample}}={\mathrm{Absorbance}\;}_{\mathrm{sample}}-{\mathrm{Absorbance}\;}_{\mathrm{sample}\;\mathrm{blank}}$.

#### 2.6.6. Lipase Inhibitory Activity

The lipase inhibitory assay was performed according to the method described by Ulpathakumbura et al. [7] and Alakolanga et al. [15], with minor modifications. A concentration series ranging from 7.18 to 1000 mg L^−1^ was prepared by dissolving the pure compound in distilled water. Firstly, 100 µL of phosphate buffer (pH 7.4) and 25 µL of sample solution were dispensed into a 96-well microplate. Subsequently, enzyme solution (50 µL) was added and incubated for 15 min at 37 °C. Thereafter, a portion of p-NPB working solution (25 µL) was added and further incubated for 30 min at 37 °C. The absorbance was recorded at 400 nm. Orlistat (Orslim tablet) was used as the positive control. The percentage of lipase inhibition was calculated using the following equation, where IC_50_ values were obtained by plotting the inhibition percentage against the respective sample concentrations:

$$Percentage\;lipase\;inhibition=\frac{\delta {A\;}_{\mathrm{control}}-\delta A_{\mathrm{sample}}}{\delta {A\;}_{\mathrm{control}}}\times 100$$

where $\delta {A\;}_{\mathrm{control}}={\mathrm{Absorbance}\;}_{\mathrm{control}}-{\mathrm{Absorbance}\;}_{\mathrm{control}\;\mathrm{blank}},$ $\delta A_{\mathrm{sample}}={\mathrm{Absorbance}\;}_{\mathrm{sample}}-{\mathrm{Absorbance}\;}_{\mathrm{sample}\;\mathrm{blank}}$.

## 3. Results

Fractionation and purification of the three extracts (hexane, EtOAc, and MeOH) yielded twelve pure isolates. The preliminary analysis indicated that compounds SAC 1, SAC 2, SAC 3, SAC 5, SAC 6, SAC 8, SAC 10, SAC 11, and SAC 12 were primarily fatty acids, plant sterols, and minor hydrocarbon fragments of limited significance. Consequently, further detailed investigations focused on compounds SAC 4, SAC 7, and SAC 9. The yields of SAC 4, SAC 7, and SAC 9 were 7.4 mg, 4.4 mg, and 3.1 mg, respectively. However, as the R_f_ values of these three compounds were similar in TLC, subsequent characterization was continued using SAC 4.

### 3.1. Structure Elucidation of SAC 4

**Figure 1:**
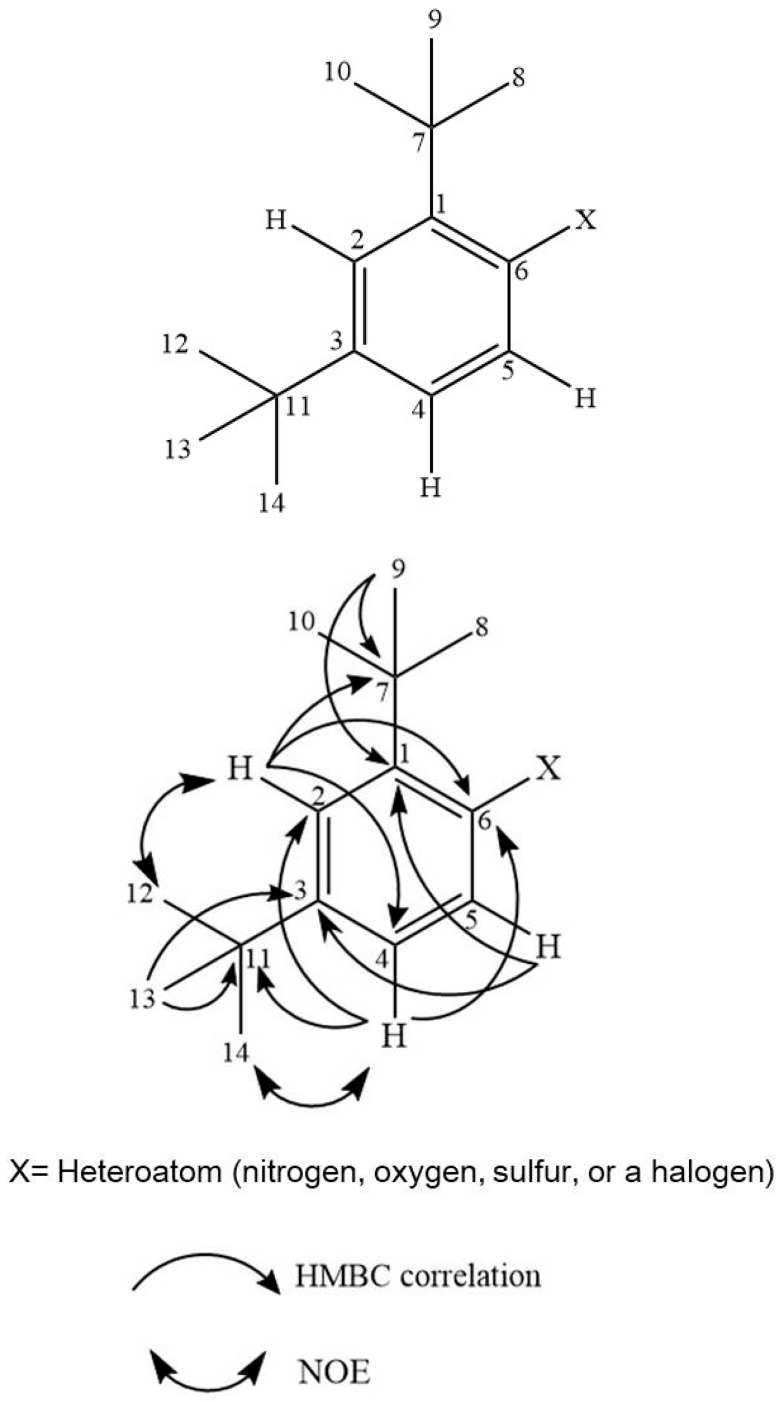
Structure of compound SAC 4 as elucidated by NMR analysis.

The ^1^H NMR and ^13^C NMR data of compound SAC 4 are presented in Table 1. The spectral data indicated the presence of five types of protons within the chemical shift range of ẟ 1.29–7.54 ppm. The 9H singlets observed at ẟ 1.33 and 1.29 ppm were attributed to two non-equivalent tert-butyl groups. Aromatic proton signals observed at ẟ 7.13, 7.36, and 7.53 ppm suggested the presence of a benzene ring. The signal at ẟ 7.13 appeared as a doublet of doublet (dd) with coupling constants of 8.8 and 2.2 Hz, while the signal at ẟ 7.54 appeared as a doublet (d) with a coupling constant of 8.8 Hz. The signals at ẟ 7.13 and ẟ 7.53 were therefore assigned to ortho-coupled protons. Additionally, the signal at ẟ 7.36 appeared as a triplet with a coupling constant of 2.2 Hz, indicating meta coupling with the proton at ẟ 7.13 (Figure S1). These arrangements were further supported by HMBC correlations (Figure S5), which confirmed the positions of the proton signals as illustrated in Figure 1.

Based on the ^13^C NMR data, signals at ẟ 31.42, 34.51, 34.85, and 30.16 ppm were assigned to the carbon atoms of tert-butyl groups (Figure S2). The positions of these groups on the benzene ring were further confirmed by HMBC correlations and NOE experiments (Figures S3–S5). Additionally, three carbon signals at ẟ 138.50/138.42 (C-1), 147.64/147.59 (C-6), and 119.07/119.06 (C-5) ppm appeared as paired signals, although the spectra were otherwise identical (Figures S2 and S3). No additional proton and carbon signals were observed. The carbon resonance of 147.64/147.59 ppm (C-6) suggested the unidentified substituent (“X”) may be associated with a heteroatom, such as nitrogen, oxygen, sulfur, or a halogen. To further clarify the nature of this substituent, FTIR spectroscopic analysis was conducted next.

### 3.2. FTIR Spectroscopy of SAC 4

**Figure 2:**
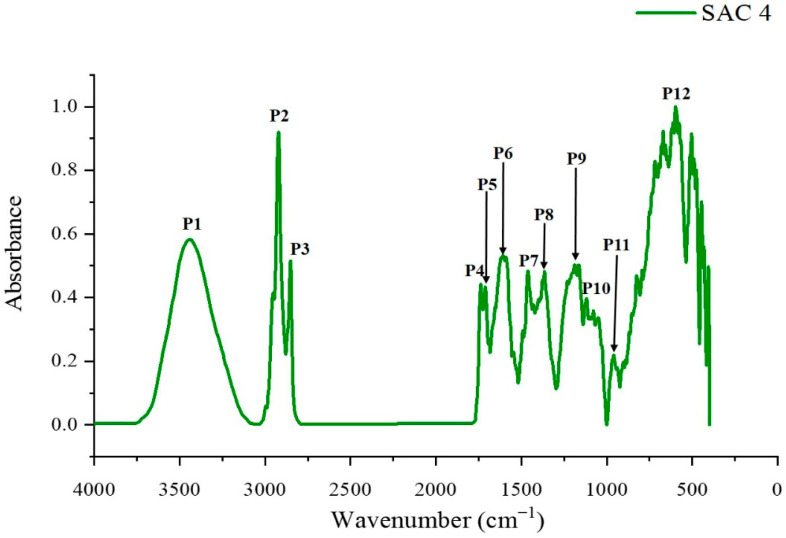
FTIR spectrum of compound SAC 4.

The FTIR spectrum of SAC 4 (Figure 2) revealed several characteristic absorption bands. A broad peak (P1) at approximately 3440 cm^−1^ is typically attributed to O-H stretching vibrations, indicating the possible presence of hydroxyl groups, such as in alcohols and phenols [16,17]. Although an -OH group was not clearly identified in the NMR data, the FTIR results suggest that the unidentified substituent (“X”) may involve a hydroxyl functionality. Two prominent peaks at ~2928 cm^−1^ (P2) and ~2854 cm^−1^ (P3) correspond to asymmetrical and symmetrical C-H stretching vibrations of methylene groups, respectively, indicating the presence of aliphatic (alkane) moieties. A peak at 1742 cm^−1^ (P4) is characteristic of C=O stretching in ester functional groups, while the peak at 1715 cm^−1^ (P5) is indicative of carbonyl stretching in carboxylic acids or ketones [16,17]. Although these functional groups were not evident in the NMR data, the FTIR spectrum suggests that the “X” substituent may involve an ester, carboxylic acid, or ketone functionality. The band at 1610 cm^−1^ (P6) corresponds to C=C stretching vibrations in aromatic rings, confirming the presence of an aromatic system. This is further supported by the peak at 1410 cm^−1^ (P7), which is associated with aromatic ring vibrations and aliphatic C-H bending [16,17,18]. Additional peaks (P8 and P9) further support the presence of both aromatic and aliphatic components in the molecule. The absorption band in the range of 1054–1120 cm^−1^ (P10) is attributed to the C-O stretching vibration of alkyl-substituted ethers, suggesting that the “X” substituent may include an ether functionality. The peak at 966 cm^−1^ (P11) is typically associated with trans-C-H out-of-plane bending of alkenes, indicating a possible unsaturated structure. Finally, the broad absorption in the region of 900–600 cm^−1^ (P12) corresponds to C-H out-of-plane bending in aromatic rings and O-H out-of-plane bending in alcohols, further supporting the presence of an aromatic system and suggesting the possible involvement of a hydroxyl group in the “X” substituent.

**Figure 3:**
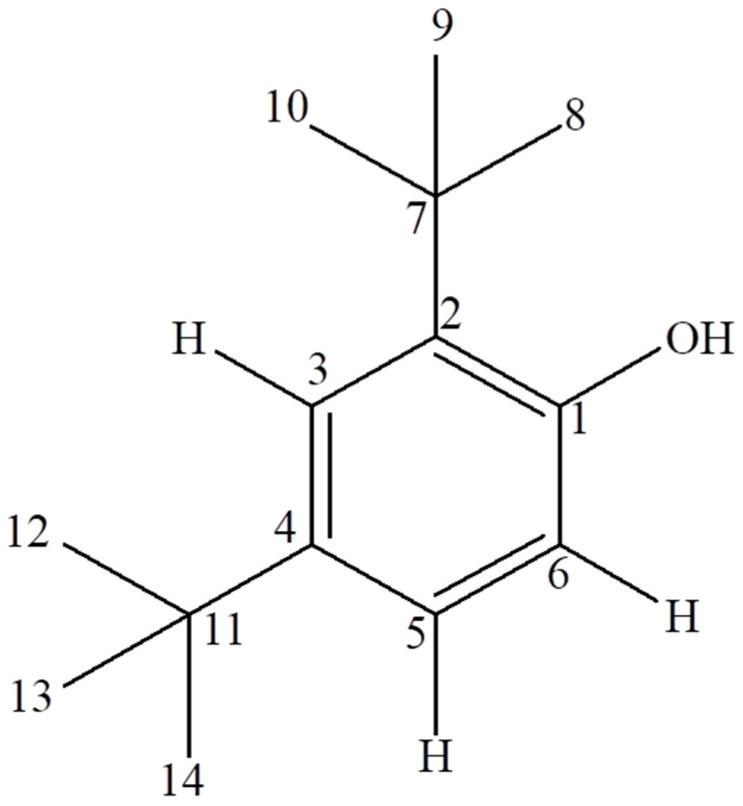
Structure of compound SAC 4 (2,4-di-tert-butylphenol).

Based on detailed analysis of ^1^H and ^13^C NMR spectra, along with key HMBC and NOE correlations and supporting FTIR data (broad O–H stretching band), the structure of compound SAC4 was identified as 2,4-di-tert-butylphenol (Figure 3).

### 3.3. Bioactivity Assessment of SAC 4

#### 3.3.1. Antioxidant Capacity

##### DPPH Radical Scavenging Activity

The data presented in Table 2 show the percentage of DPPH radical scavenging activity of the compound SAC 4 at different concentrations. SAC 4 displayed a relatively low DPPH radical scavenging activity, with a maximum of 41.16 ± 0.56% at 100 mg L^−1^. Accordingly, the IC_50_ value for SAC 4 is estimated to be higher than 100 mg L^−1^. In contrast, ascorbic acid (positive control) demonstrated significantly stronger activity, with an IC_50_ value of 8.39 ± 0.05 mg L^−1^.

##### ABTS Radical Scavenging Activity

The results of the ABTS assay are presented in Table 3. This assay evaluates the capacity of antioxidant compounds to neutralize the ABTS•+ stable radical cation. Consistent with DPPH results, SAC 4 exhibited low ABTS radical scavenging activity, with a value of 138.09 ± 1.91 µmole Trolox equivalents per gram of sample. In comparison, ascorbic acid showed substantially higher activity (1783.17 ± 2.62 µmole Trolox equivalents per gram of extract).

##### Ferric Reducing Antioxidant Power (FRAP)

The FRAP assay is also shown in Table 3. The assay measures the reduction of ferric ions (Fe^3+^) to ferrous ions (Fe^2+^) under acidic conditions [19]. In line with the DPPH and ABTS findings, SAC 4 demonstrated low ferric reducing power (0.07 ± 0.01 µmole FeSO_4_ equivalents per gram of sample). In contrast, ascorbic acid exhibited significantly higher reducing capacity (10,316.26 ± 19.20 µmole FeSO_4_ equivalents per gram of extract).

### 3.4. Enzyme Inhibitory Activity

#### 3.4.1. α-Glucosidase Inhibitory Activity

**Figure 4:**
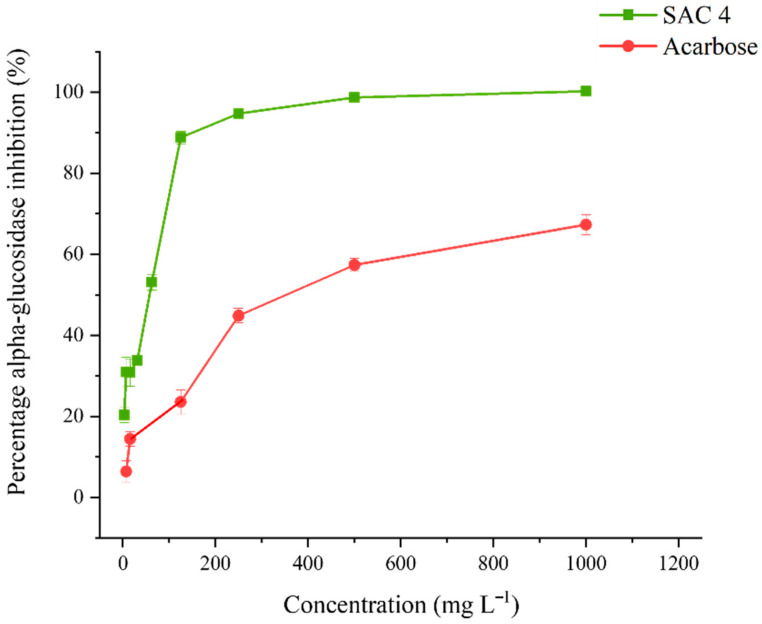
Percentage of α-glucosidase inhibition of compound SAC 4, with acarbose as the positive control.

The percentage of α-glucosidase inhibitory activity and IC_50_ values of SAC 4 are presented in Figure 4. Acarbose (positive control) exhibited an IC_50_ value of 386.00 ± 16.03 mg L^−1^. Notably, SAC 4 demonstrated significantly stronger α-glucosidase inhibitory activity, with a much lower IC_50_ value of 55.56 ± 1.25 mg L^−1^, indicating higher potency compared to the standard drug.

#### 3.4.2. Lipase Inhibitory Activity

**Figure 5:**
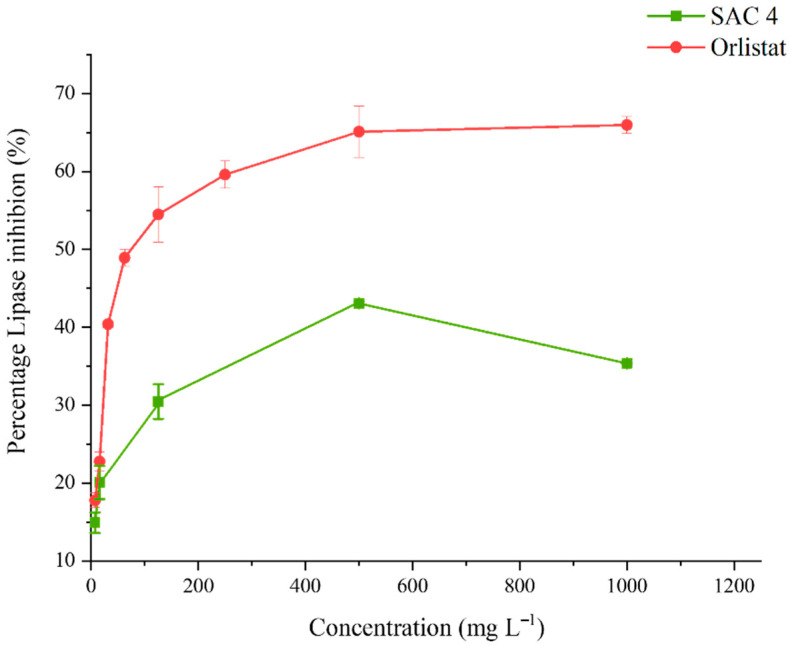
Percentage of lipase inhibition of compound SAC 4 using Orlistat as a control.

The percentage of lipase inhibition of compound SAC 4 is presented in Figure 5. At a concentration of 1000 mg L^−1^, SAC 4 exhibited moderate lipase inhibitory activity (35.37 ± 0.08%). In contrast, Orlistat (positive control) demonstrated substantially higher inhibitory activity, with an IC_50_ value of 63.74 ± 1.70 mg L^−1^.

## 4. Discussion

### 4.1. Secondary Metabolites of A. ceylanica

Reports on the isolation of secondary metabolites from the leaves of *A. ceylanica* are relatively limited; however, several earlier studies provide some insights. Fraser & Lewis [20,21] first isolated two alkaloids, atalanine and ataline, from the woods of *A. ceylanica*. Subsequently, Ahmad et al. [22] and Murray & Hall [23] reported the isolation of ceylantin from the heartwood. Bowen & Patel [24] later isolated four compounds from the leaves of *A. ceylanica*, including two acridone alkaloids (5-dihydroxy-3-methoxy-10-methyl-9(10H)-acridinone and 11-hydroxynorac-ronycine), 2,4,5-trimethoxy-benzaldehyde, and the pyranoflavone carpachromene. In addition, Bacher et al. [25] isolated two isomeric aldoximes, ataloxime A and ataloxime B, from the seeds of *A. ceylanica*, which exhibited insecticidal activity against *Spodoptera littoralis* larvae. The seed extracts were also reported to contain higher levels of heraclenin, furanocoumarins, bergapten, imperatorin, xanthotoxin, and oxypeucedanin [25]. Despite these findings, previous studies on leaf-derived metabolites have provided limited information regarding their biological activities. Therefore, the present study aimed to evaluate the biological potential of purified compounds from *A. ceylanica*, particularly in terms of anti-diabetic, antioxidant, and anti-obesity activities.

### 4.2. Biological Activities

#### 4.2.1. Phytonutrient Profile

The distribution of selected phenolic compounds in *A. ceylanica* leaves has been previously reported by Ulpathakumbura et al. [7]. Among various leafy plant extracts, *A. ceylanica* exhibited the highest total phenolic content (TPC) in both hexane and EtOAc extracts. LC-MS analysis identified nine compounds: caffeic acid, catechin, caffeine, ferulic acid, chlorogenic acid, p-coumaric acid, gallic acid, rutin, sinapic acid, and vanillin. Among these, rutin was the most abundant, followed by sinapic acid [7]. Rutin is well recognized for its powerful antioxidant properties, along with anti-inflammatory, neuroprotective, and cardiovascular effects. Similarly, sinapic acid, commonly found in fruits, vegetables, and cereals, exhibits antioxidant, antiglycemic, anticancer, anti-inflammatory, antimutagenic, neuroprotective, and antibacterial potentials [26]. Earlier work by Fernando & Soysa [11] also confirmed the presence of phytoconstituents such as phenols and flavonoids in *A. ceylanica*, which are widely associated with antioxidative properties in plant-based foods. Furthermore, Dharmadasa et al. [27] reported that leaf extracts of *A. ceylanica* may contain essential oils such as caryophyllene, caryophyllene oxide, α-cardinol, decanal, and lauraldehyde, and demonstrated antimicrobial properties.

#### 4.2.2. Antioxidant Activity

Antioxidants play a crucial role in maintaining oxidative balance in the human body by neutralizing free radicals generated during metabolic processes. The results of the DPPH, FRAP, and ABTS assays indicated moderate antioxidant activity for the leaves of *A. ceylanica*. Previous studies reporting radical scavenging activities (DPPH, FRAP, and ABTS) of *A. ceylanica* leaves are limited. However, Ulpathakumbura et al. [7] demonstrated that the antioxidant activity of crude leaf extracts, assessed using FRAP and ABTS assays, is influenced by the extraction solvent. Among different leafy plant extracts, ABTS^+^ radical scavenging activity was observed in the methanol extracts of all plant types, whereas for *A. ceylanica*, ABTS radical scavenging activity was detected only in the ethyl acetate extract. Furthermore, the highest FRAP value among the tested samples was reported for the ethyl acetate extract of *A. ceylanica* [7].

#### 4.2.3. Enzyme Inhibitory Activity

α-amylase and α-glucosidase are key enzymes involved in carbohydrate digestion and are crucial in regulating postprandial hyperglycemia [8,13]. Although *A. ceylanica* leaves have traditionally been used for treating liver and respiratory conditions, their antihyperglycemia potential has been scarcely investigated. The findings of the present study suggest that consumption of this leafy plant may contribute to glycemic control, as its major isolate, SAC 4, exhibits significant α-glucosidase inhibitory activity. Previous investigations on crude extracts of *A. ceylanica* leaves revealed that *α*-amylase inhibitory activity (in EtOAc extracts) and *α*-glucosidase inhibitory activity (in hexane extracts) were positively correlated with antioxidant properties, including FRAP, DPPH, and ABTS activities [7].

Pancreatic lipase is the key enzyme responsible for lipid digestion and is a major target in the development of anti-obesity therapeutics [28]. Inhibition of this enzyme is a recognized strategy for reducing fat absorption. In previous studies, the lipase inhibitory activity of five leafy plants was generally low and dependent on both plant type and extraction solvent [7]. Orlistat, a well-known lipase inhibitor, irreversibly inhibits gastrointestinal lipases and was found in this study to exhibit significantly higher activity (*p* < 0.05), with an IC_50_ value of 63.74 ± 1.70 mg L^−1^, compared to plant extracts. In contrast, the pure isolate of SAC 4 showed relatively low lipase inhibitory activity (29.47 ± 1.15%), indicating limited potency in this regard. Precious studies have reported potent lipase inhibitory activity in extracts from sources such as *Phellinus* mushrooms, *Acanthopanax sessiliflorus* leaves, and *Flacourtia inermis* fruits [15,29,30].

## 5. Conclusions

Chromatographic separation and purification of crude extracts of *A. ceylanica* (YK) yielded twelve compounds, of which three (SAC 4, SAC 7, and SAC 9) were present in appreciable quantities. Of these, the chemical structure of SAC 4 was elucidated using ^1^H and ^13^CNMR data along with FTIR spectroscopy. Bioactivity assays revealed that SAC 4 exhibits strong α-glucosidase inhibitory activity, moderate antioxidant capacity, and moderate lipase inhibitory activity. These findings suggest that SAC 4 has potential as a bioactive compound for managing hyperglycemia. However, further studies are required to fully confirm its chemical structure and evaluate its potential for development as a plant-based therapeutic agent for diabetes treatment.

## Figures and Tables

**Table 1:** ^1^H NMR (500 MHz, CDCl_3_) and ^13^C NMR (125 MHz, CDCl_3_) of compound SAC 4.

C/H Position	ẟH (Number of H, Multiplicity, J in Hz)	ẟC, Type
1	-	138.50/138.42, C
2	7.36 (1H, t, 2.2)	124.45, CH
3	-	147.05, C
4	7.13 (1H, dd, 8.8,2.2)	123.97, CH
5	7.53 (1H, d, 8.8)	119.07/119.06, CH
6	-	147.64/147.59, C
7	-	34.85, C
8	1.33 (3H, s)	30.16, CH_3_
9	1.33 (3H, s)	30.16, CH_3_
10	1.33 (3H, s)	30.16, CH_3_
11	-	34.51, C
12	1.29 (3H, s)	31.42, CH_3_
13	1.29 (3H, s)	31.42, CH_3_
14	1.29 (3H, s)	31.42, CH_3_

**Table 2:** Percentage of radical scavenging activity of the SAC 4 compound ^1^.

Concentration (mg L^−1^)	Percentage of Radical Scavenging Activity
0.78	12.64 ± 1.26
1.5625	27.74 ± 2.43
3.125	29.81 ± 2.09
12.5	32.77 ± 0.42
50	35.44 ± 0.56
100	41.16 ± 0.56

^1^ Each value in the table represents the mean of the three replicates ± standard deviation.

**Table 3:** FRAP and ABTS radical scavenging values of SAC 4 ^1^.

**Compound**	**Ferric Reducing Antioxidant Power** **(µmole of FeSO_4_ per g of Sample)**
SAC 4	0.07 ± 0.01
Ascorbic acid (control)	10,316.26 ± 19.20
**Compound**	**ABTS radical scavenging activity** **(µmole of Trolox g^−1^ of sample)**
SAC 4	138.09 ± 1.91
Ascorbic acid (control)	1783.17 ± 2.62

^1^ Each value in the table represents the mean of the three replicates ± standard deviation.

## Data Availability

The data that support the findings of this study are available from the corresponding author upon request.

## Supplementary Materials

The following supporting information can be downloaded at: https://www.mdpi.com/article/10.3390/biom16050663/s1, Figure S1: SAC 4,7,9-PTLC-3-^1^H-500M-CDCL_3_; Figure S2: SAC 4,7,9-PTLC-3-^13^C-125M-CDCL_3_; Figure S3: SAC-4,7,9-DEPT135-125-CDCL_3_; Figure S4: SAC-4,7,9-PTLC-3-COSY-500M; Figure S5: SAC-4,7,9-PYLC-3-HMBC.
