# Supplementary material for: Characterization of a Pure Isolate from Atalantia ceylanica Leaves and Its Biological Activities
Source: Biomolecules. 2026 Apr 30;16(5):663. doi: 10.3390/biom16050663 (PMC13204049; doi:10.3390/biom16050663)

Supplementary Information

NMR Spectrums of Isolated SAC 4 Compound

Figure S1: SAC 4,7,9-PTLC-3-^{1}H-500M-CDCL$_3$

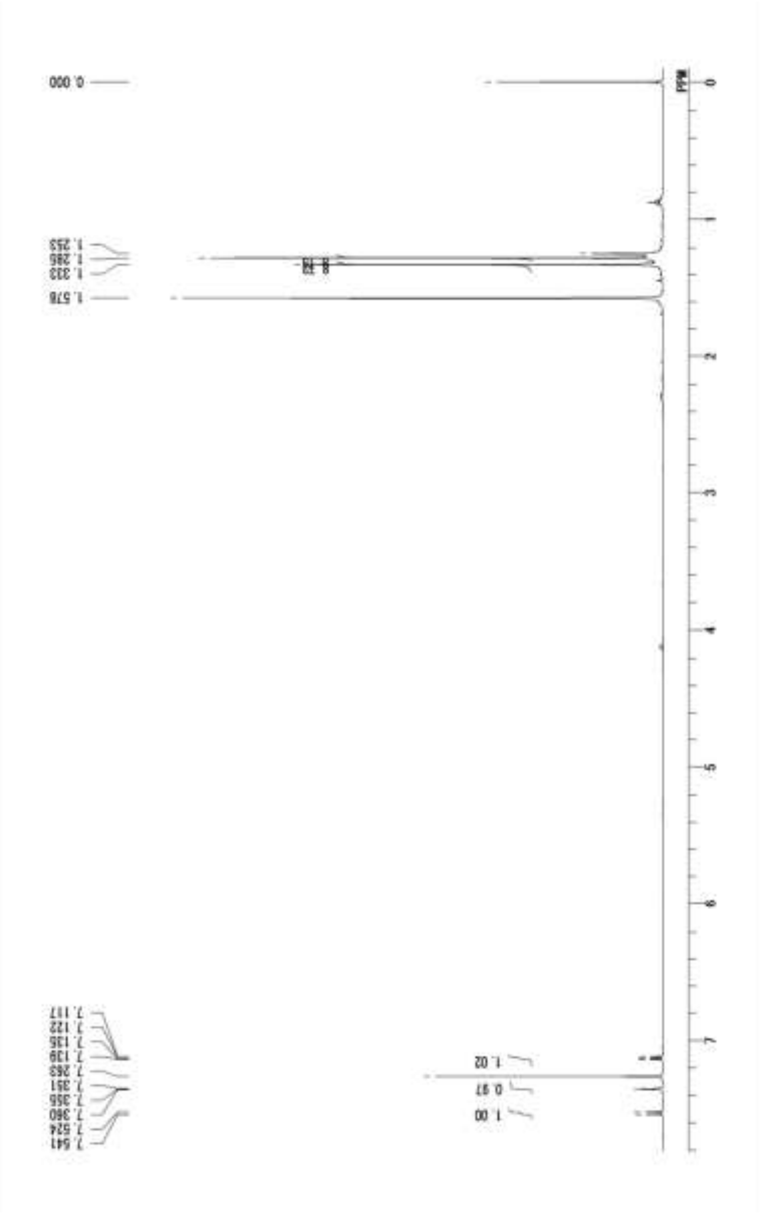

Figure S2: SAC 4,7,9-PTLC-3-^{13}C-125M-$CDCL_3$

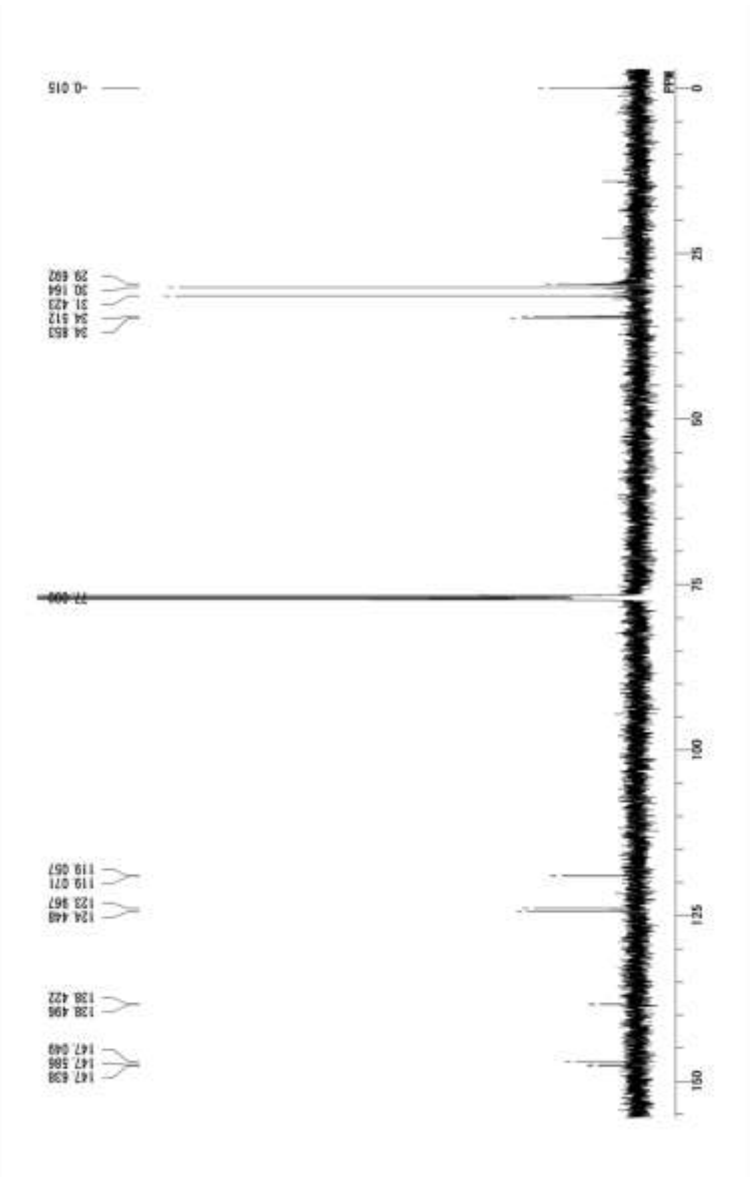

Figure S3: SAC-4,7,9-DEPT135-125-CDCL$_3$

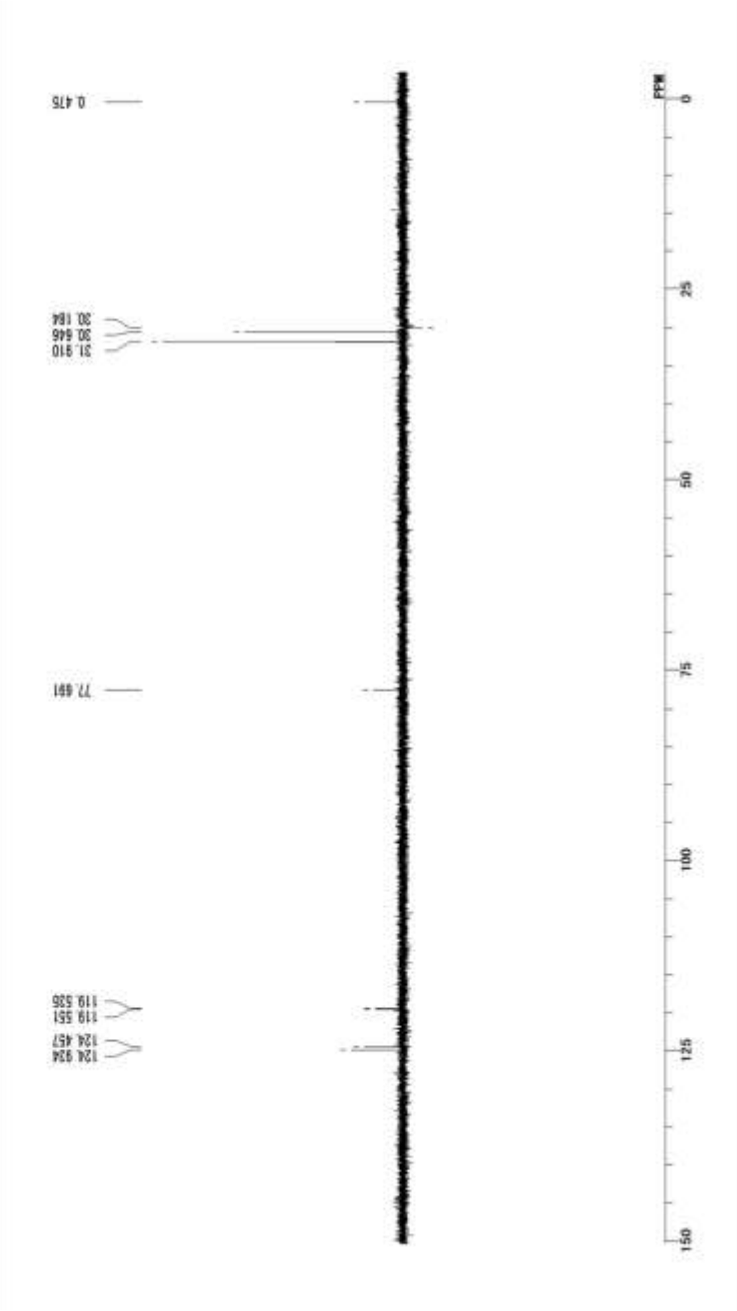

Figure S4: SAC-4,7,9-PTLC-3-COSY-500M

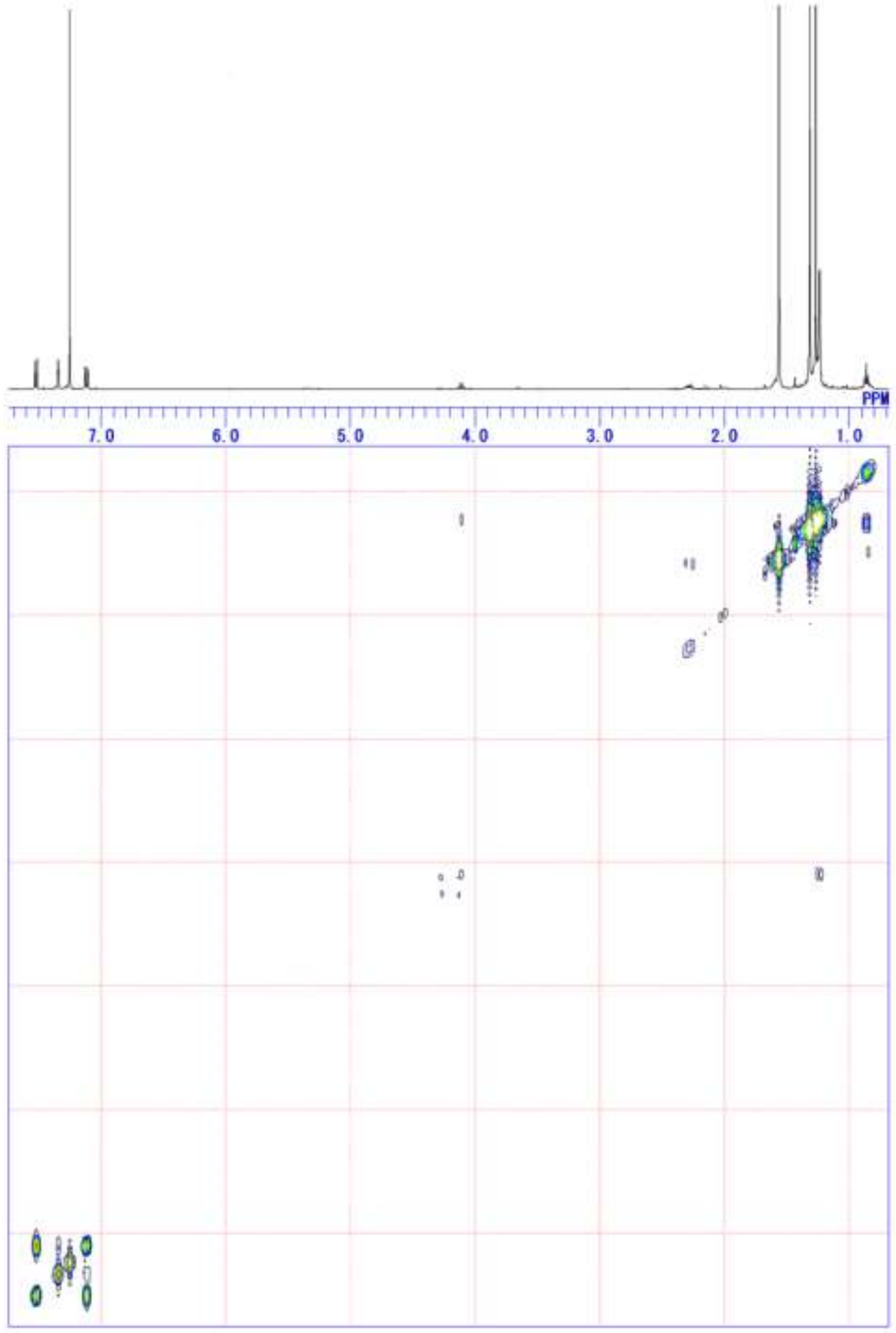

Figure S5: SAC-4,7,9-PYLC-3-HMBC

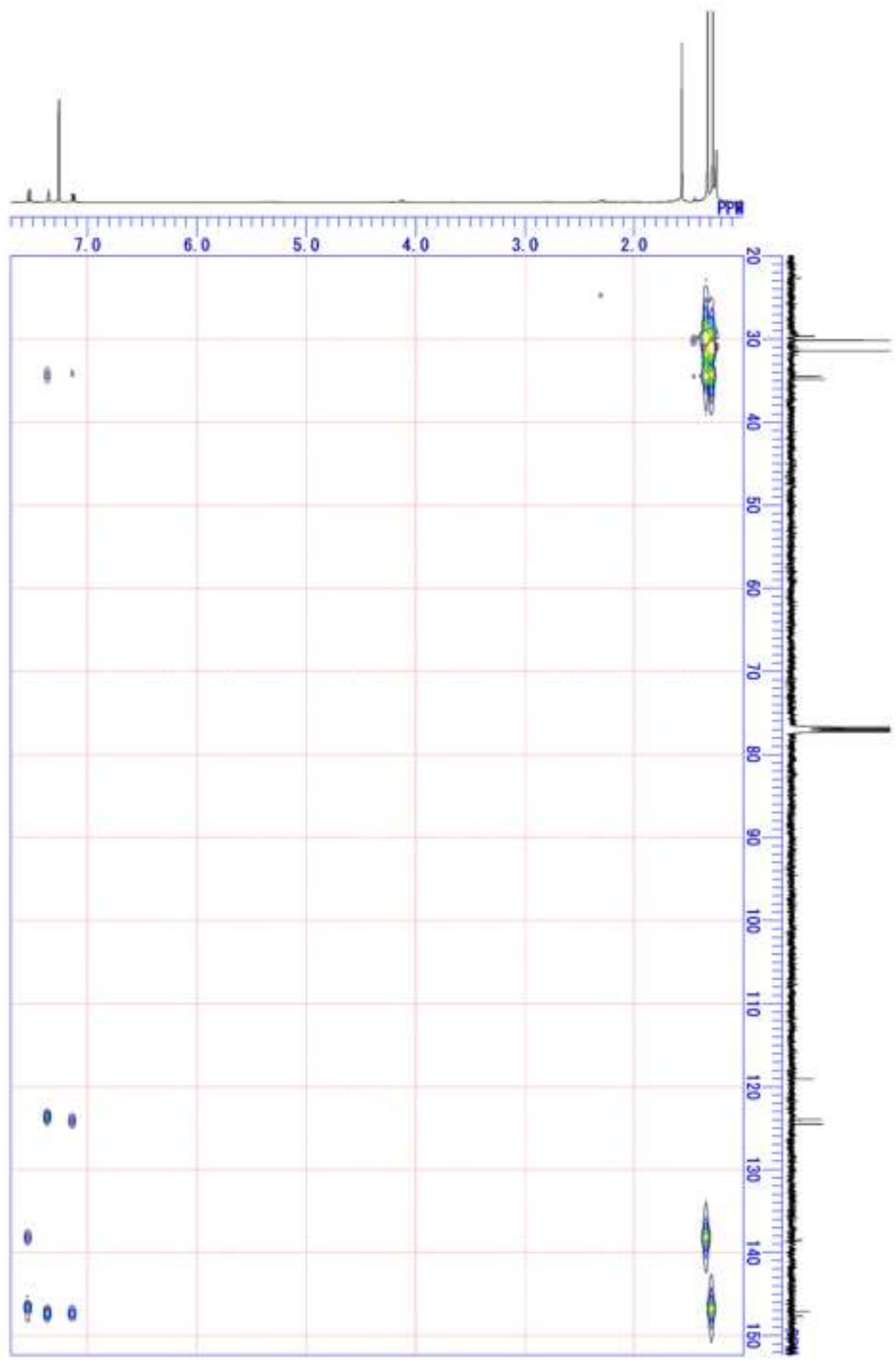

Supplement: Supplementary file 1 [file biomolecules-16-00663-s001.zip › biomolecules-4236896-supplementary.pdf]
